# CACONET: Ant Colony Optimization (ACO) Based Clustering Algorithm for VANET

**DOI:** 10.1371/journal.pone.0154080

**Published:** 2016-05-05

**Authors:** Farhan Aadil, Khalid Bashir Bajwa, Salabat Khan, Nadeem Majeed Chaudary, Adeel Akram

**Affiliations:** 1 Department of Computer Engineering, University of Engineering and Technology, Taxila, 45020, Pakistan; 2 Department of Computer Science, COMSATS Institute of Information Technology, Attock, 43600, Pakistan; Beihang University, CHINA

## Abstract

A vehicular ad hoc network (VANET) is a wirelessly connected network of vehicular nodes. A number of techniques, such as message ferrying, data aggregation, and vehicular node clustering aim to improve communication efficiency in VANETs. Cluster heads (CHs), selected in the process of clustering, manage inter-cluster and intra-cluster communication. The lifetime of clusters and number of CHs determines the efficiency of network. In this paper a Clustering algorithm based on Ant Colony Optimization (ACO) for VANETs (CACONET) is proposed. CACONET forms optimized clusters for robust communication. CACONET is compared empirically with state-of-the-art baseline techniques like Multi-Objective Particle Swarm Optimization (MOPSO) and Comprehensive Learning Particle Swarm Optimization (CLPSO). Experiments varying the grid size of the network, the transmission range of nodes, and number of nodes in the network were performed to evaluate the comparative effectiveness of these algorithms. For optimized clustering, the parameters considered are the transmission range, direction and speed of the nodes. The results indicate that CACONET significantly outperforms MOPSO and CLPSO.

## Introduction

Clustering is a technique for assembling a group of nodes (mobile gadgets, devices, automobiles, etc.) inside a geographical locality according to certain regulations. Such regulations vary from one algorithm to another and, therefore, are the decisive aspect in creating dependable clusters [1]. Clusters are virtual sets created using a clustering algorithm. Each cluster is composed of cluster nodes (CN), which nominate or elect a single CH. The group of nodes within a CH’s transmission range is referred to as its neighborhood. In most cases, any CN can be elected as the CH; however, in several algorithms, some types of nodes possess more effective properties for becoming the CH. For instance, a CN with a supplemental 3G network connection is often more desirable than its non-3G peers [2–4]. Cluster size depends on the nodes’ transmission range, and as a result varies from cluster to cluster [4–6].

**Fig 1:**
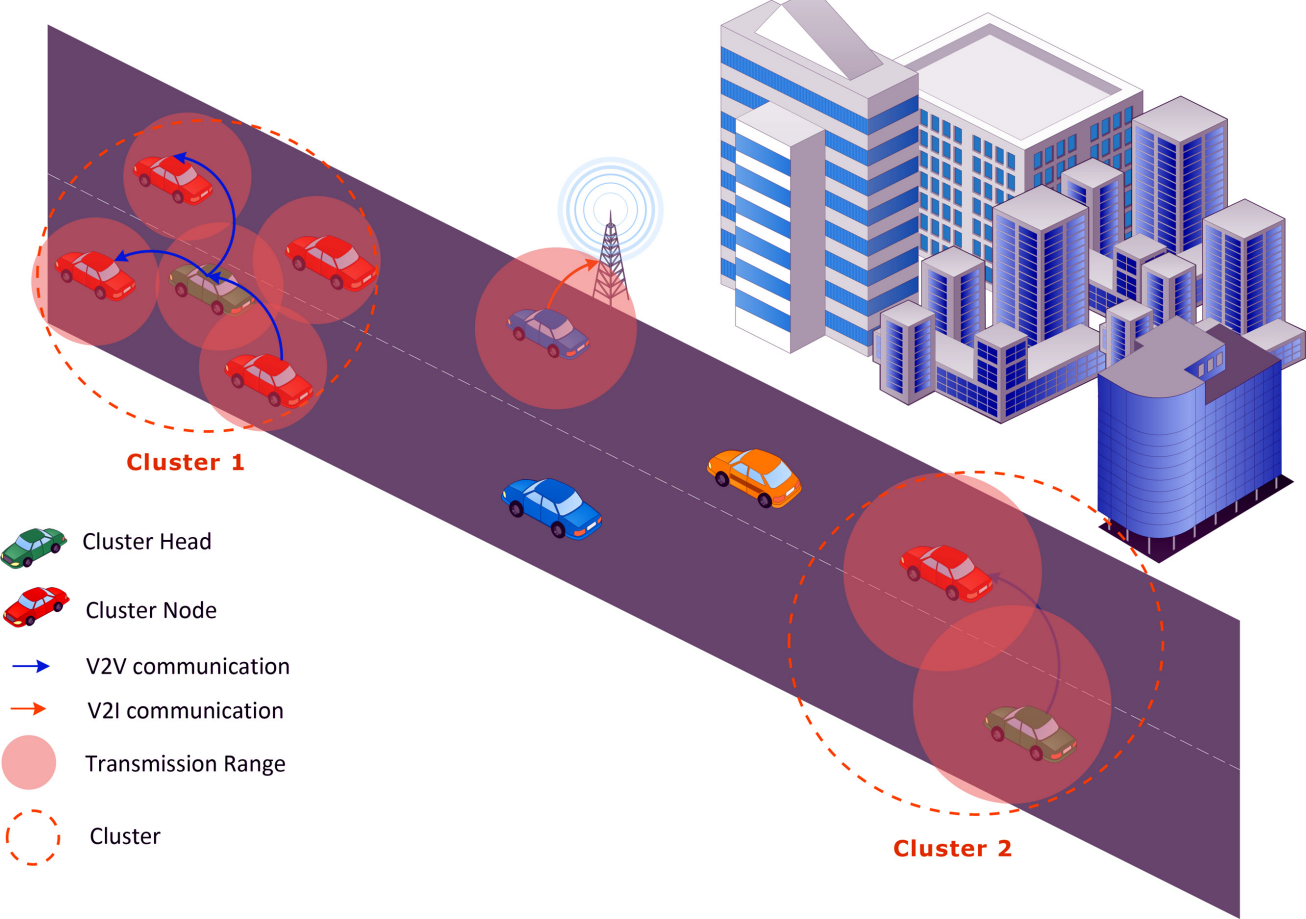
Clustering in VANET.

Ad hoc networks are a vibrant research area, and VANET is a type of mobile ad hoc network (MANET) that transforms automobiles on the roads into network nodes. These nodes create a dispersed network of automobiles for information exchange [7]. The potential applications for VANETs include safety, comfort, and infotainment related applications [8]. One type of VANET communication is vehicle-to-vehicle communication (V2V), an ad hoc mode that operates in the 75 MHz Dedicated Short Range Communications spectrum. Along with one control channel, there are six service channels in this spectrum. The topology of VANET changes rapidly due to a very high-mobility node pattern. Even though a node’s mobility is predictable, VANET’s lifetime is difficult to extend. Scalability is an essential issue in VANETs and one solution is clustering; clustering is important for load balancing and efficient resource utilization, and it helps to optimize the network and to make it more scalable. Clustering entails segregating the network into small logical groups, as shown in Fig 1, which increase the lifetime of the network. MOBIC [3] is one of the most frequently referenced clustering algorithms, and it focuses solely on MANETs. Relative to MANETs and sensor ad hoc networks, VANETs are a more recently proposed network type and, as a result, they are an under explored area of research and extensive efforts are still needed to develop the field. Several research studies [9–11] explain the differences between these three types of networks (i.e., VANET, MANET, and sensor ad hoc networks) and their respective challenges.

Clusters in which any pair of nodes can either communicate directly or with one hop are referred to as 1-hop clusters [6]. In this type of cluster, every CN can send messages directly to its CH, and two CNs can easily "talk" with one another, either directly or through their CH. Convenience is the primary motive for the use of 1-hop clusters. Other solutions utilize greater than one-hop communication, and these are termed n-hop clusters. Cluster stability is a key feature of clustering algorithms, and a way of measuring their effectiveness. The cluster stability is important for the upper and lower communication layers, and can raise their performances significantly [12]. It simplifies routing, permits spatial reuse of resources, and helps the network to appear more stable to the CNs. The most frequently used parameters of cluster stability are i) the number of CH changes and ii) the number of CNs switching their CH. By diligently picking the CH along with the CNs that form a specific cluster, the cluster’s stability is improved considerably [1]. The CH is responsible for forming the cluster, maintaining the network topology, and distributing resources to all nodes in the cluster. Due to dynamic nature of VANET, the topology changes very fast and, therefore, the CH’s configuration changes frequently. In this scenario, it is necessary to minimize the number of CHs. The optimal selection of CHs is an NP-hard problem [13].

There are prerequisites associated with clustering in VANETs. The clustering algorithms need to be dispersed, since every node within the network possesses only local knowledge and, due to cluster-based routing, communicates out of the cluster via its CH. The algorithm must be robust as, if the network grows or shrinks, and/or there are any other changes in the network, it needs to adjust to these transformations. The clusters need to be quite effective, i.e., the determined CHs should handle as many nodes as possible.

### Literature Review

Several of the principal techniques proposed by researchers for optimizing network efficiency are discussed in this section. In a clustering algorithm presented in [14], a unique ID number is assigned to each node and at any time, the node with the lowest ID is chosen as the CH. A highest-connectivity clustering algorithm is proposed by Gerla and Tsai [2]. This algorithm is a multi-cluster, multi-hop packet radio network architecture for wireless adaptive mobile information systems. In this scheme neighbors of a given node are initially identified by calculating the node degree. Each node announces its identifier for the election procedure. Once the degree is computed, the node with the highest degree becomes the CH. In [5] the authors proposed a clustering algorithm to optimize the number of clusters for ad hoc networks. In the Weighted Clustering Algorithm (WCA) [10], a weight is assigned to each objective by the user. It was one of the first clustering algorithms developed for MANETs. In this algorithm CHs are elected according to their weight. The weights are calculated by combining different parameters. The CH selection process is non-periodic to reduce the communication and computation costs and the call to CH selection procedure is on demand. The diameter of the basic network is directly proportional to the time required to identify the CHs. A clustering algorithm based on n-hops for MANET is proposed in [6]. In this technique the diameter of the cluster is flexible and not restricted to two hops, and the clusters are formed on the basis of similar node movement patterns. In [15] a clustering scheme is derived mathematically; the parameters of network connectivity, average velocity difference, relative velocity, and average distance are take into consideration in this scheme.

One real-world influenced, evolutionary approach uses swarm intelligence. This technique is employed to resolve a variety of challenging optimization problems. It follows the structure of an insect swarm: many individual insects are contained in an insect nest, and while an individual insect is not a highly intelligent being, the communal entity can form a collective intelligence. For example, when bees maintain the temperature in the hive, local stimuli are responded to by every reactive agent (insect) without any central reasoning. This swarm behavior is evident in social insects such as ants, wasps, bees and termites. Using the artificial bee colony algorithm, a dynamic node clustering technique is proposed in [16]; variants of random and greedy selection are used in this technique.

Beneath the umbrella of swarm intelligence there are a couple of key clustering algorithms, such as ACO and particle swarm optimization (PSO). PSO is an algorithm proposed by [17], inspired by the maneuvering of a flock of birds. In this algorithm, every individual in the flock is guided according to the best personal and best global behavior, which converges them into a near optimal geographical position. For MANETs, Shahzad et al. [18] proposed a Comprehensive Learning Particle Swarm Optimization (CLPSO) based clustering algorithm. This algorithm finds the optimal number of clusters in a network, and each particle (solution set) contains the information for its CH and CN(s). The algorithm assigns a weight to network parameters like battery power consumption, node transmission power, node mobility, and ideal degree.

The basic parameters for MANETs are not considered in any of these heuristic-based algorithms [18–20]. The WCA does not find the ideal number of clusters in the network; however, it was one of the first algorithms to include the maximum number of parameters (including the ideal node-degree, transmission power, mobility and the battery power of the nodes). WCA provides a single solution by making a multi-objective problem a single-objective problem through assigning weight to each objective function [10]. Multiple solutions are provided by the MOPSO based clustering algorithm [19].

In multi-objective optimization problems (MOPs), evolutionary algorithms are a reliable way of obtaining multiple solutions. These algorithms are designed to get several solutions at a time rather than just a single solution. Many evolutionary algorithms have been developed to work with different mechanisms, for instance, the genetic algorithm [21], differential evolution, artificial immune system, and swarm intelligence [17, 18, 20, 22–26], among others. ACO based techniques for MOPs are also documented in the literature. Most of these documented algorithms are only applicable to problems where a lexicographic ordering of the objectives is provided, for instance, where the objectives can be listed according to their significance [27, 28].

ACO is one of the best metaheuristics, and it constructs a graph of the optimization problem. This graph is then explored by artificial ants for the best possible solution to the given problem [29]. Initially each ant finds its local solution and then lays pheromone trails over the search space to encourage other ants to further explore the surroundings of the best solutions found. The successful implementation of evolutionary algorithms for optimized clustering [5, 11, 18, 30] encouraged us to develop an ACO based algorithm named CACONET.

### Clustering as an Optimization Problem

Optimization challenges are highly significant to scientific engineering models and other decision-making applications. Optimization is the discovery of several solutions for a problem, which correspond to the extreme values connected with more than one objective. When an optimization problem has just one objective, the task of choosing the best possible solution is referred to as a single-objective problem. With the exception of multimodal functions, the focus in a single-objective problem is typically on obtaining just a single solution. MOPs are optimization problems that come with several objective functions. The majority of real-world problems are MOPs, as they encompass a variety of objectives that have to be optimized concurrently. Clustering in VANET is an example of a MOP [30]. Many conventional mathematical programing approaches produce a single solution for MOPs. For that reason, such approaches may not be appropriate for the optimization of MOPs. Evolutionary algorithm paradigms are preferable for MOPs, as they are population based, which enables them to produce multiple solutions in a single iteration [22], as follows:

$$f=W1(f_{1}(d))+W2(f_{2}(d))+W3(f_{3}(d))+\cdots Wn(f_{n}(d))$$ (1)

**Fig 2:**
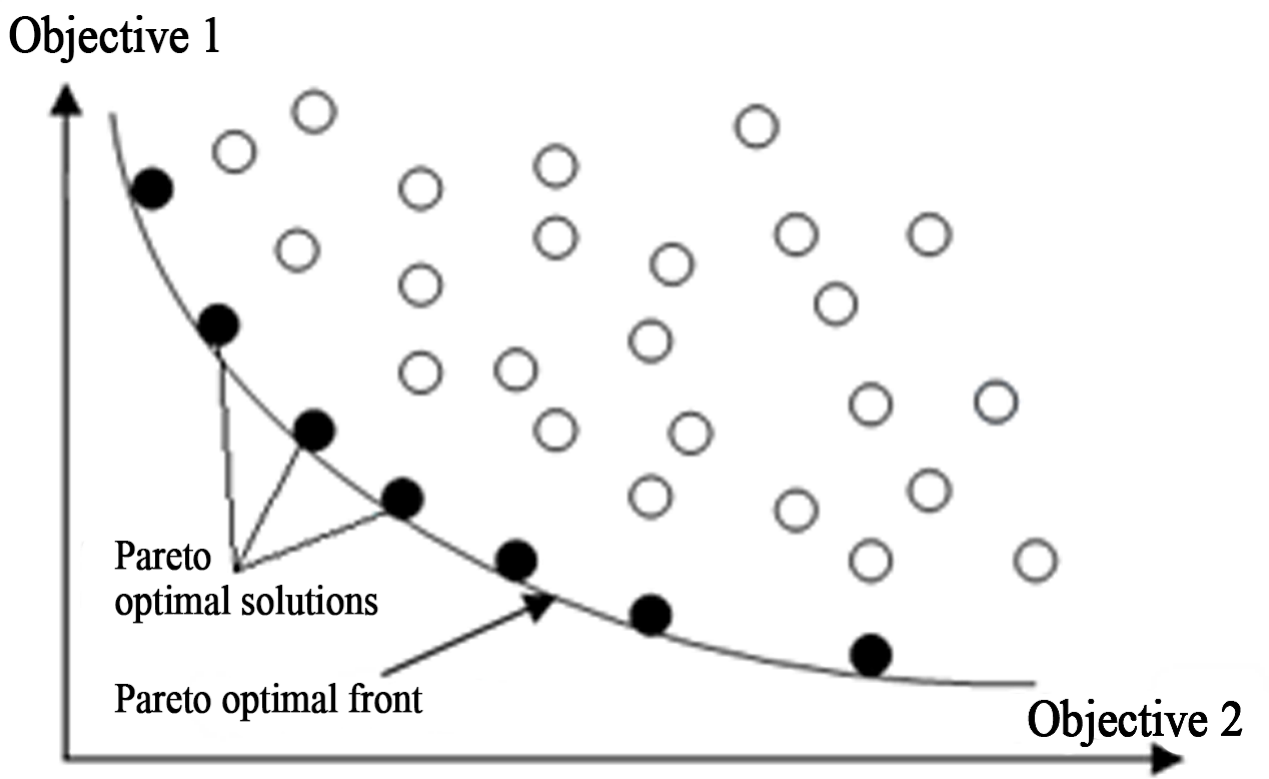
Non-dominated solutions for two contradictory objective optimization problems.

MOPs contain numerous desired goals which are minimized or perhaps maximized at the same time [19]. Such problems possess numerous specifications that a solution must satisfy. The search space is multidimensional in multi-objective optimization. Suppose there are *n* objective functions: *f*_1_(d), *f*_2_(d),…, *f*_n_(d). The final evaluation *f* of a solution is based on the weighted summation of these objective functions as given in Eq 1, where W*i* represents the weight assigned to *i*^th^ objective function in the range 0 to 1, and d represents the decision variables. As an example, decision variables for clustering in VANET are: 1) the distance of neighboring nodes from the CH (the less, the better); 2) the speed of the CH and the CNs (the more similar, the better); and 3) the direction of CH and CNs in a cluster (the more similar, the better). It is possible that more than one optimal solution is found for the same values of *f*. The variable d* is called the Pareto optimal solution (decision variables) when there is no possible vector of decision variable d ∈ D that will reduce some objective value(s) while not increasing any other objective value(s) at the same time (meaning the final *f* value remains the same). In most cases, this specific strategy produces a group of solutions, known as the Pareto optimal solutions. The curve joining these non-dominated solutions is called a Pareto optimal front [23]. All solutions on a Pareto optimal front are labeled Pareto optimal solutions. For instance, Fig 2 shows two objective functions that are contradictory with one another. As multi-objective clustering is the focus of the proposed technique, two objective functions (delta difference and distance of CH from CNs) of the VANET environment, with equal weights, are utilized in Eq (1) for this purpose.

There are two search spaces in MOPs, one is the decision variable space and the other is the objective space. The range is specified within these spaces. Multiple Pareto optimal solutions are found only if contradictory objectives exist in *f*. If the objectives are not contradictory with one another, then there will be just one search space (the decision variable space). However, there are two search spaces in MOPs, and for this reason MOPs are considered challenging.

#### Clustering via PSO

In PSO, each solution to the problem is called a particle. The particles combined are referred to as a swarm and the swarm is used to find a near optimal solution. Suppose $\vec{X_{i}}$ is the position vector for a particle *i*, the dimensions of the $\vec{X_{i}}$ vector are equal to the number of parameters/attributes in the problem. $\vec{P_{i}}$ is the position of its personal best solution, and $\vec{V_{i}}$ is its velocity at this point. The local best solution is known to each particle. Particle positions and velocities are initially generated randomly and then updated iteratively. In each iteration of the algorithm, the new positions and velocities are calculated as follows:

$$V_{id}={WV}_{id}+(p_{id}-x_{id})(c_{1})(r_{1})+(p_{gd}-x_{id})(c_{2})(r_{2})$$ (2)

$$x_{id}=V_{id}{+x}_{id}$$ (3)

Where *W* is the inertia weight; *i* = 1, 2,…, N, for a population size N; r_1_ is the first random number generated by a uniform distribution in the interval [0, 1]; and *r*_2_ is the second random numbers generated by an uniform distribution in the interval [0, 1]. The variable *d* = 1, 2,…, D, where D is the maximum number of iterations. The variables *c*_1_ and *c*_2_ are first and second positive constants respectively. For the *i*^th^ particle, current velocity is computed using Eq (1). This is done while considering three terms: i) the particle’s best personal position, ii) the global best position, and iii) the particle’s previous velocity.

The new position of a particle is calculated using Eq (2). Inertia weight is introduced to control the impact of the previous velocities on the current velocity. If inertia weight is eliminated, it means there is no previous history of a particle’s velocity. To ignite the process, PSO is initialized with a collection of random solutions, or particles, after which PSO explores for the best possible solutions in each generation. In each iteration, each particle updates its personal best value achieved and the global best position obtained by any particle in the population up until that time.

### CACONET: An ACO Based Clustering Algorithm for VANET

A VANET is made more stable with an optimal number of clusters because the network resources are efficiently utilized. In this scenario, for instance, the job of routing network packets within the cluster or to the nodes of other clusters can be done by the CHs alone rather than by each node in the cluster. The evolutionary capability of ACO enables our proposed algorithm to optimize the number of clusters in the network.

In ACO based techniques, one solution is called an ant and the group of ants form a swarm, which looks for the best solution. These techniques work very efficiently and are suitable for continuous and discrete variable problems. Although their implementation is comparatively difficult, these techniques are computationally inexpensive, especially when compared to the situation in which an exhaustive search to identify the best solution is performed. These features mean that ACO based techniques are very effective for clustering in ad hoc networks, especially in VANETs. CACONET is the first attempt to achieve efficient clustering in VANETs using ACO. The algorithm initially finds the CH, and then neighborhood for this CH.

The ACO metaheuristic usually models the real-world environment of ants in the form of a graph. The vertices of the graph represent the components of a candidate solution. Ants traverse the edges to create trails. While traversing different paths, ants mark the route taken with a chemical substance called a pheromone. The artificial pheromone values are associated with the edges and updated based on the quality of the trail. The higher the quality of a trail, the higher the concentration of the pheromone, and this makes the trail more attractive to the ants. An artificial ant constructs a candidate solution to the problem by adding solution components one by one. Before the construction of a complete candidate solution, a problem dependent heuristic is usually used in collaboration with the pheromone values to guide the ants’ movements. Subsequently, as time passes, ants construct their solutions one by one and guide each other to find better and better solutions. The components with higher pheromone concentrations are thus identified as contributing to a good solution and repeatedly appear in the solutions. Usually, after sufficient iterations, the ants converge towards a very good, if not the optimal, solution.

The application of ACO to a problem requires the following (28):

- The ability to represent a complete solution as a combination of different components.
- A method to determine the fitness or quality of the solution.
- A heuristic measure for the solution’s components (this is desirable but not essential).

The pseudo code for CACONET is presented in Table 1 and the major stages of the proposed algorithm are discussed below.

**Table 1:** Proposed CACONET algorithm.

Pseudo code for proposed CACONET algorithm
*1:* Initialize all vehicles’ positions randomly on the highway
*2:* Randomly initialize each vehicle’s direction
*3:* Initialize the speed/velocity of each vehicle
*4:* Create a mesh topology among nodes/vertices, where each vertex represents a vehicle ID
*5:* Initialize the same pheromone values for each edge in the above mesh topology
*6:* Calculate the distance of each vehicle from the others, normalize and associate these distance values with the corresponding edges in the above mesh topology
*7:* **WHILE** (Iteration = = Total Iterations OR Stall Iteration = = 20) (no improvement in last 20 Iterations)
*8*: {
*9:* **FOR** Ant_i_ = 1 to Swarm size
*10:* Ant_i_.tour = = empty, and cost = = infinity
*11:* Vertices or Nodes–Available for clustering = {All Nodes}
*a:* **WHILE** (Nodes Available for clustering! = empty)
*b:* **END WHILE**
*c:* Ant_i_.cost = evaluation (Ant_i_.tour)
**IF** (Ant_i_.cost < Best Ant.cost)
Best Ant = Ant_i_
*d:* Ant_i_ ++
*e:* **END FOR**
*12:* **FOR** Ant_i_ = 1 to Swarm size
*i.* Update Pheromone (Ant_i_.tour, Ant_i_.cost)
*a.* Evaporate
*b.* **IF** (BestAnt.cost = = Last iteration Best.Ant.cost)
*ii.* Stall Iteration ++;
*c.* **ELSE**
*iii.* Stall Iteration = 0;
*d.* **END IF**
*e.* Iteration ++;
*13:* **END WHILE**
*14:* CHs = Best Ant.tour;

#### Search Space Construction

The ACO algorithm based solution to a particular problem starts with the design of a problem search space in which the ants conduct the search to find the candidate solutions. The search space for CACONET is a mesh topology based graph as described in Table 1. The labels of the vertices in the graph represent the IDs of vehicles/nodes in the VANET. For example, to perform clustering of a VANET environment with 30 vehicles, the search space will consists of 30 vertices each connected via mesh topology. The edges between the vertices are associated with two values: 1) pheromone value, and 2) heuristic value. In the subsequent subsections, more detail is provided about these two values.

#### Pheromone Initialization

The edges in the search graph are initialized with low pheromone values. The initial pheromone *τ*_*ij*_ over the edge between two vertices *i* and *j* is laid down based on the following equation:

$$\tau_{ij}\;(iter=1)=\frac{1}{\left|Vehicles\right|}$$ (4)

Where |Vehicles| represents total number of the vehicles in the network.

#### Solution Construction

In each iteration of the FOR loop (line #9) of the algorithm in Table 1, each ant constructs its solution. An ant starts its tour by randomly selecting a vertex in the search space. Later, the ant selects and incorporates more vertices into its tour, taking into consideration pheromone and heuristic values over the edges subject to some constraints. The vertices in an ant tour are the CHs for clustering. So, each ant tour is a collection of CHs for the given VANET environment. The constraints for the selection of a vertex to be incorporated into the tour are given as:

1. A vertex can only be added to the tour if it is not already present in tour. This constraint makes it sure that a vehicle cannot be selected as CH more than once in a tour/solution. The tour consists of uniquely labeled vertices that represent the CH vehicles in the VANET.
2. A vertex cannot be added into the tour if it is in the transmission range of a vertex already present in the tour. Once a CH is selected, all the vehicles in the transmission range of the CH become a member of the cluster. This constraint ensures that a cluster is controlled by only one CH.

In the proposed algorithm, the probability of a vertex (from the search space) being added into the tour of the current ant is calculated using Eq 5:

$$P_{i,j}=\frac{{Pheromone}_{i,j}\times {Heuristic}_{i,j}}{\sum_{k\in S}{Pheromone}_{i,k}\times {Heuristic}_{i,k}}$$ (5)

Where *i* is the label of the vertex last added into the tour of the current ant, *j* is the label of next candidate vertex which can be selected by the ant, and *P*_*i*,*j*_ is the selection probability of the edge between vertices *i* and *j*. *S* is the set of all vertices available for selection subject to the two constraints detailed above. *Pheromone*_*i*,*j*_ and *Heuristic*_*i*,*j*_ are pheromone and heuristic values associated with edge between vertices *i* and *j*, respectively. The selection probability of an edge is divided by the sum of the selection probabilities of all the edges available for traversal. The higher the pheromone and heuristic values of an edge, the better its chances of selection are. In order to make sure that the algorithm doesn’t become stuck in local optima, the selection of an edge is performed by roulette wheel selection [31]. In other words, the edge with lowest selection probability still has a chance of selection and the selection of edge is not based on greed. Once an edge is selected, the current ant moves over the edge and reaches a new vertex in the search space. So, the selection of an edge is actually the selection of next vertex to be added to the tour of the current ant.

The tour of an ant is completed when the above-mentioned constraints mean that there are no more vertices available to be added to the tour. It is important to note that the tour lengths are variable. A tour with a lower number of CHs or clusters is usually preferable as this lowers the communication overhead.

#### Evaluation of Solution and Heuristic Value Calculation

The tour/solution of an ant is then evaluated to determine its worth. Due to the multi-objective nature of VANET clustering, the following modified version of Eq 1 is used to evaluate the tour of ant *t*:

$$f_{t}=W1(f_{1})+W2(f_{2})$$ (6)

Where *W*1 = *W*2 = 0.5 represents the equivalent weights assigned to two objective functions *f*_1_ and *f*_2_, respectively. For CACONET, *f*_1_ is the delta difference value of the clusters in *t*, and *f*_2_ is the summation of the distance values of all CHs from their cluster members. The delta difference value *d* of the clusters in a tour can be calculated by employing Eq 7:

$$d=\sum_{i=1}^{\left|t\right|}ABS(D-|{CN}_{i}|)$$ (7)

Where *D* is a constant value and represents the ideal degree of clusters. The value of *D* is assigned by the user. For example, if the user needs dense clusters, *D* may be assigned a high value and vice versa. |*t*| is the length of the tour or, in other words, the total number of clusters formed. |*CN*_*i*_| is the total number of vehicles in cluster *i*, excluding CH. The ABS function returns the absolute value of the given value. The lowest value of *d* represents the formation of clusters almost equivalent to the user-specified ideal degree. If value of *d* is zero, the clustering is optimal in terms of the user’s ideal degree requirements.

The value for objective function *f*_2_ can be calculated based on the Euclidean distance (ED) between the cluster members and the CHs for all the clusters. Distance between the CH and all of its member nodes can be calculated using Eq 8:

$${dist}_{CH\_i}=\sum_{j=1}^{\left|{CN}_{i}\right|}ED({CH}_{i},{CN}_{j,i})$$ (8)

Here CH_i_ represents the coordinate position of the *i*^th^ CH. CN_*j*,*i*_ is the coordinate position of the *j*^th^ CN which is the member of cluster *i*. Similarly, the *f*_2_ objective value is calculated using Eq 9:

$$f_{2}=\sum_{i=1}^{\left|t\right|}{dist}_{CH\_i}$$ (9)

Again, |*t*| is the tour length or, in other words, the total number of clusters. Similar to *f*_1_, the lowest possible value for *f*_2_ is preferable. The shorter the distance between CH and its cluster members, the less the energy will be required to transfer the data.

Having discussed solution/tour construction, a discussion of the heuristic value calculation over an edge follows here. Suppose the ant is over vertex i and has to calculate the heuristic value over the edge between vertex i and j; Eq 6 can be used for this purpose. Eq 6 is used for evaluating the completed tour; however, the same equation is also used for the heuristic calculations for an incomplete tour (i.e., there are still vertices available that can be added into tour). For incomplete tours, every single available vertex is added in the tour, one at a time, and its worth is calculated using Eq 6. In this way, the available vertices are assigned heuristic values in accordance with their worth as determined by Eq 6.

#### Update Pheromone in Search Space

Pheromone values on the edges are an important learning dynamic for the CACONET. To make efficient use of pheromone values, the quality of ant tours is employed. The pheromone values on the edges constituting the trail are updated in proportion to the quality of the trail and so define the learning directions for the subsequent transitions of the entire swarm. Eq 10 is used to update the pheromone values over the edges between the vertices in the trails constructed by ants.

$$\tau_{ik}\;(t+1)=(1-\rho )\tau_{ik}\;(t)+(1-\frac{1}{1+f_{n}}\tau_{ik}\;(t)$$ (10)

Where *τ*_*ik*_*(t)* is the pheromone value encountered in iteration *t* of the outer most WHILE loop (line #7, Table 1) between *vertex*_*i*_ and *vertex*_*k*_. The pheromone evaporation rate is represented by *ρ* and *f*_*t*_ is the worth of the tour of the n^th^ ant.

Eq 10 updates pheromones by first evaporating a percentage of the previously seen pheromone and then adding a percentage of the pheromone depending on the quality/worth of the trail constructed by the n^th^ ant. This pheromone update is carried out for all tours constructed by all the ants. If the tour corresponds well to the clustering requirement (based on Eq 6), a greater quantity of pheromone is added than is evaporated and the vertices found in the tour become more attractive to the ants in subsequent iterations. Evaporation improves exploration; in the presence of a static heuristic function the ants tend to converge quickly on the terms selected by the entire swarm during the first few iterations of the first inner repeat loop [32].

#### Stopping Criterions

In this section, different criterions to stop the execution of the CACONET algorithm are discussed. The first criterion to stop the execution of CACONET is the completion of the total number of iterations specified by user (line #7, Table 1). The second criterion occurs when the stall iteration count reaches 20 (initially started at 0). An iteration is considered to stall if there is no improvement in the quality of best trail found in outermost WHILE loop as compared to the quality of best trail found in previous iteration of outermost WHILE loop. Finally, after stopping the execution of CACONET, the best tour found so far is used for the clustering of the VANET.

### Implementation and Results

Experimental setup is described in this section along with a comparison of the results for our performed experiments. Results from our proposed CACONET algorithm were compared with those from two other popular clustering algorithms, i.e., MOPSO [11] and CLPSO [18] based clustering. The experimental results demonstrate that the proposed technique addresses the entire network with a minimum number of clusters, which can reduce the routing cost of the network. This allows a decrease in the number of hops and packet delays in the cluster-based routing. Typically there will be more clusters when the transmission ranges of the nodes are small. The final results indicate that the proposed clustering technique is effective and adaptable in comparison to other techniques and functions more effectively than the other algorithms in a VANET environment. The algorithm optimizes the parameters associated with the vehicular nodes in order to seek the optimal solution. The parameters used in simulations are presented in Tables 2 and 3.

**Table 2:** Simulation parameters for MOPSO and CLPSO.

Parameters	Values
Population size (particles)	100
Maximum iterations	150
Inertia weight *W*	0.694
*c*_1_^1^	2
*c*_2_^1^	2
Vehicle velocity range	22 m/s—30 m/s
Simulation area	1 × 1 km^2^, 2 × 2 km^2^, 3 × 3 km^2,^ 4 × 4 km^2^
Maximum acceleration m/s^2^	1.5
Minimum distance b/w Vehicles	2 m
Maximum distance b/w Vehicles	5 m
Lane width	50 m
Total lanes	8
Transmission range	100 m–600 m
Mobility model	Freeway mobility model
Simulation runs	10
*W*1 (weight of first objective function)	0.5
*W*2 (weight of second objective function)	0.5

^1^Learning Factor

**Table 3:** Simulation parameters for CACONET.

Parameters	Values
Population size (ants)	100
Maximum iterations	150
Evaporation rate	0.05
*c*1*	2
*c*_2_*	2
Vehicle velocity range	22 m/s—30 m/s
Simulation area	1 × 1 km^2^, 2 × 2 km^2^, 3 × 3 km^2,^ 4 × 4 km^2^
Maximum acceleration m/s^2^	1.5
Minimum distance B/W Vehicles	2 m
Maximum distance B/W Vehicles	5 m
Lane width	50 m
Total lanes	8
Transmission range	100 m–600 m
Mobility model	Freeway mobility model
Simulation runs	10
*W*1 (weight of first objective function)	0.5
*W*2 (weight of second objective function)	0.5

#### Experimental Setup

MATLAB version 8.5.0 is used for implementation purposes. The experiments are conducted on a machine with 8 GB of RAM and a 2.5 GHz core i5 processor. The experiments are performed by varying the number of nodes from 10 to 60. Four sizes of road segment were used for performing these experiments: 1 km × 1 km grid, 2 km × 2 km grid, 3 km × 3 km grid, and 4 km × 4 km grid. The movement of all nodes is bi-directional along the X-axis, with velocity varying uniformly between 80 km/h (22 m/s) and 120 km/h (30 m/s). For each node the transmission range is also varied from 100 m to 600 m. For load balancing in the ad hoc network the degree difference value is set to 10. In this research, along with CACONET, two well-known evolutionary algorithms are implemented for clustering in VANET, namely CLPSO and MOPSO. All the values of different parameters are kept same for the three algorithms. Ten simulations are performed for each algorithm and their average is presented in results/graphs.Transmission Range vs Number of Clusters

**Fig 3:**
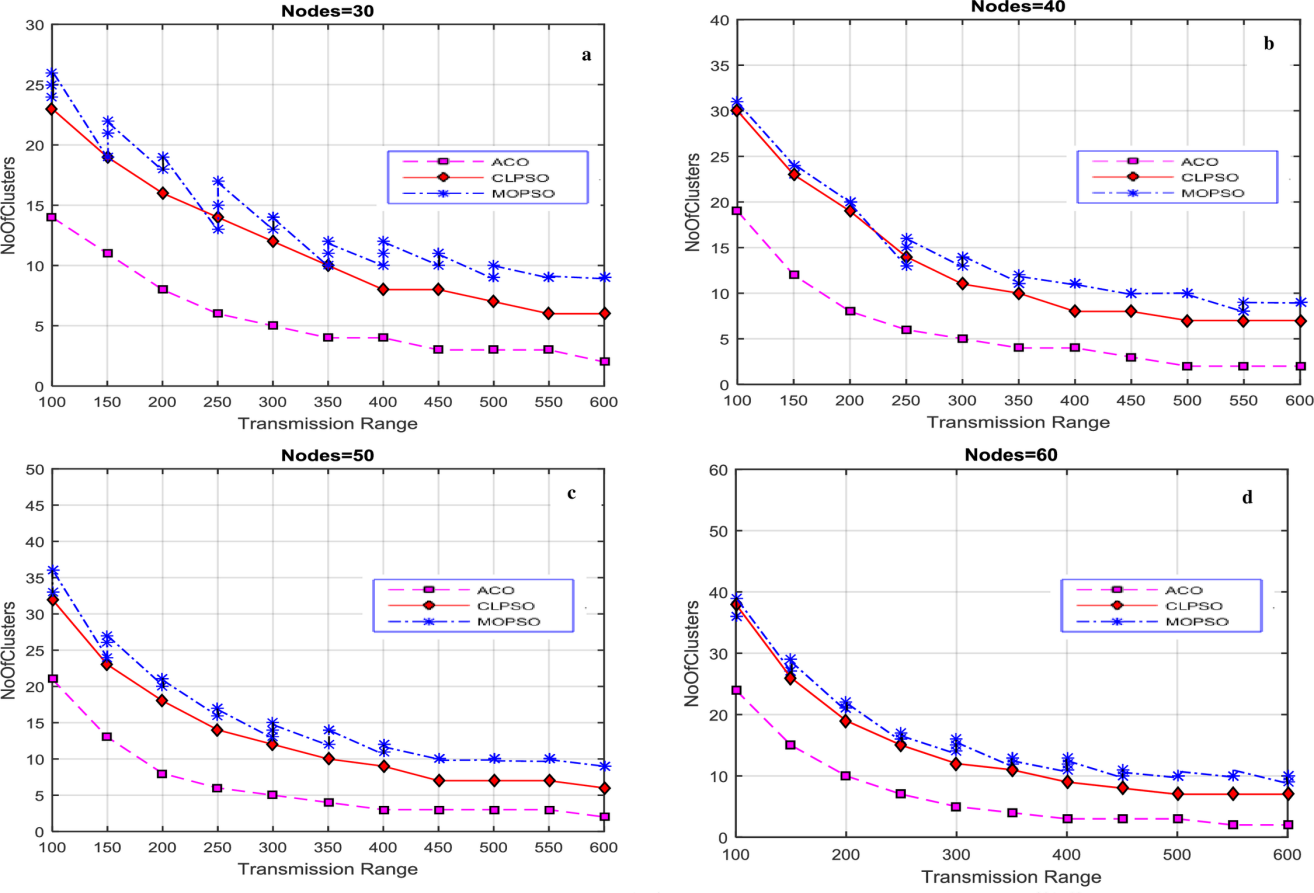
Transmission range vs number of clusters in MOPSO and CLPSO in the 1 km × 1 km grid size with nodes ranging from 30 to 60.

The transmission range of each node is varied from 100 m to 600 m and the number of nodes vary as 30, 40, 50, and 60. As a result four diverse solutions were produced. Results were generated by varying the size of road segment to 1 km × 1 km, 2 km × 2 km, 3 km × 3 km, and 4 km × 4 km. The proposed algorithm finds the optimized solutions against each transmission range, which is exhibited in Fig 3. These solutions cover the entire network, in contrast with CLPSO and MOPSO. The average number of clusters is used as a performance metric, shown in Fig 3. For the 1 km × 1 km grid size our proposed algorithm produces less clusters for each transmission range to cover the whole network, as compare with the CLPSO and MOPSO algorithms. The number of clusters produced by CACONET is less than the number produced by CLPSO and MOPSO in most cases. Although MOPSO does produce multiple solutions, the number of clusters generated by CACONET is better optimized than MOPSO.

**Fig 4:**
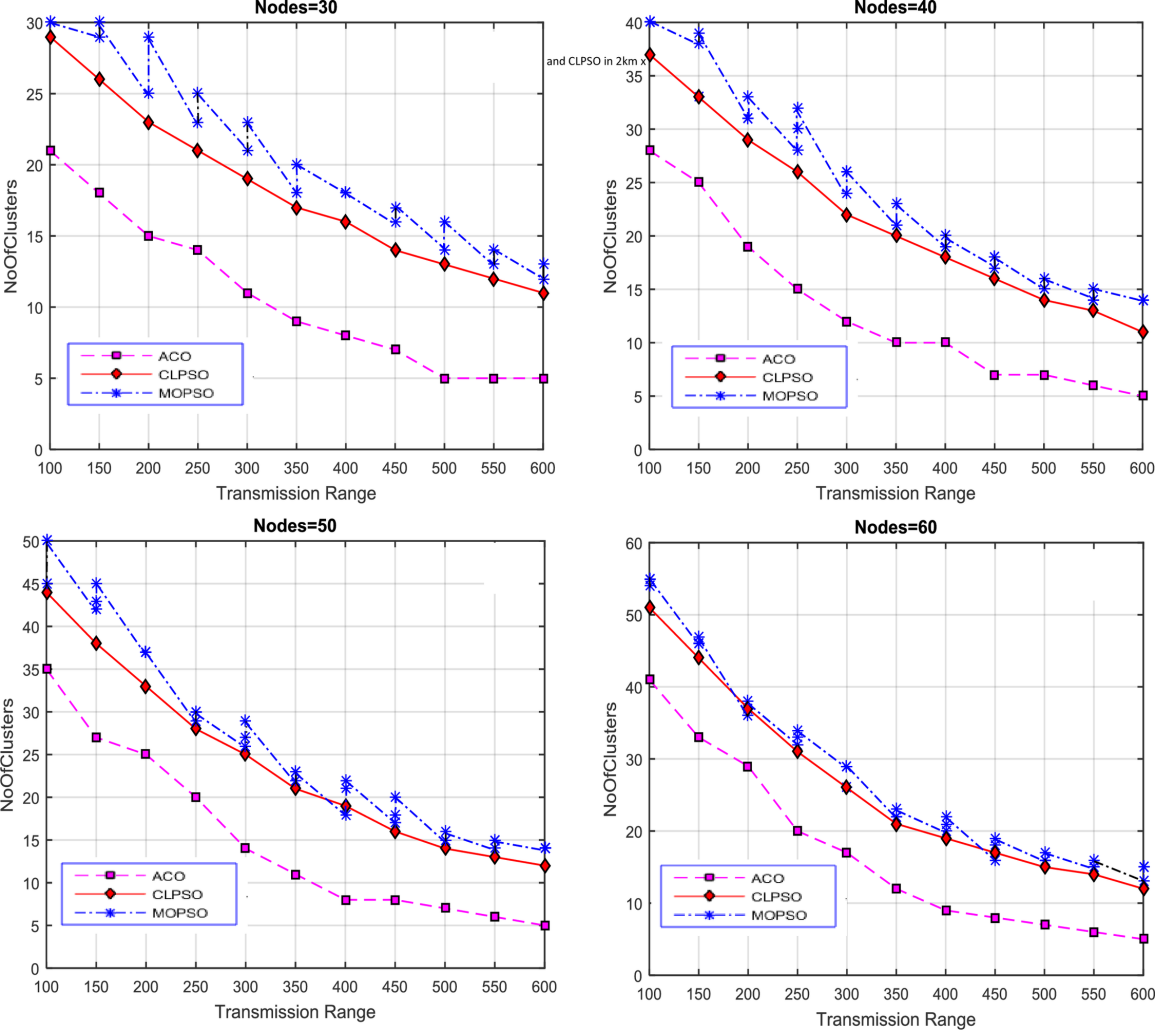
Transmission range vs number of clusters in MOPSO and CLPSO in the 2 km × 2 km grid size with nodes ranging from 30 to 60.

After these initial experiments, the size of road segment is changed to a 2 km × 2 km grid. The results of this setup are displayed in Fig 4. The results show that there are more clusters when the transmission range is low. This is because nodes are inaccessible to each other, and so there are fewer nodes in each cluster. As the transmission range rises, the number of nodes in a cluster increases, and number of clusters in each solution decreases. CACONET outperforms CLPSO in all experiments in providing improved solutions. In Fig 3(D), at transmission ranges 200 and 450, the results of MOPSO and CLPSO are almost same, but CACONET produces less clusters.

**Fig 5:**
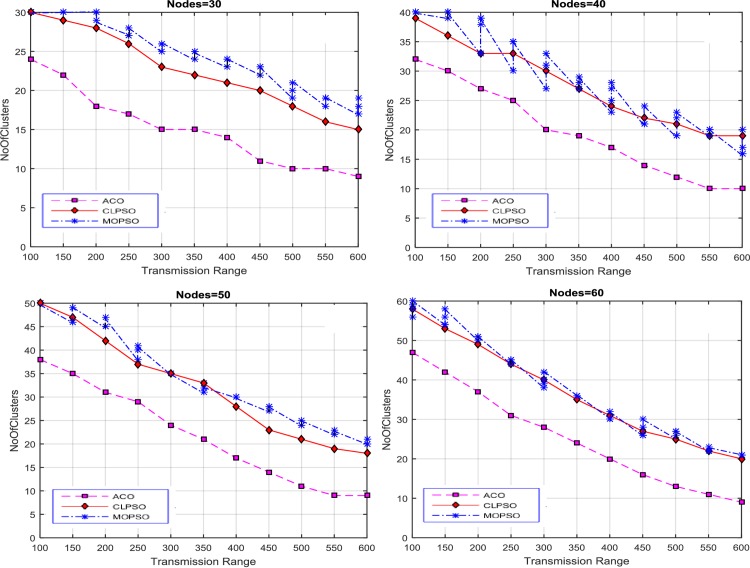
Transmission range vs number of clusters in MOPSO and CLPSO in 3 km × 3 km grid size with nodes ranging from 30 to 60.

At this point we changed the grid size to 3 km × 3 km as shown in Fig 5. The total number of clusters in Fig 5(A) is almost equal to the total number of nodes. This is because the network area is very large and the node transmission range is comparatively small. So there is direct relation between node transmission range and road segment size. It is also evident that, in the case of MOPSO, the number of solutions increases as the transmission range increases.

**Fig 6:**
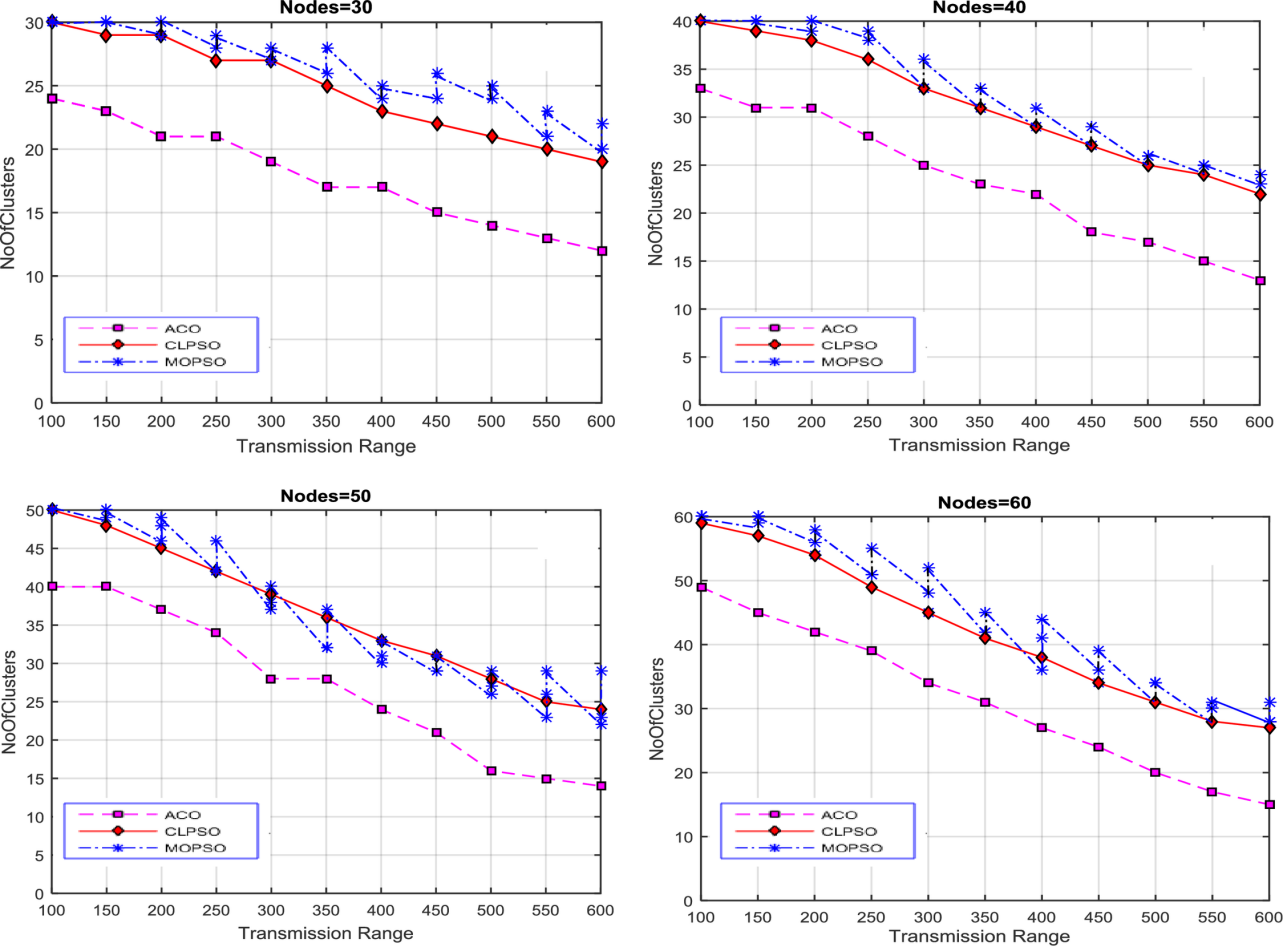
Transmission range vs number of clusters in MOPSO, and CLPSO in 4 km × 4 km grid size with nodes ranging from 30 to 60.

Now the grid size is changed to 4 km × 4 km. In Fig 6(D) MOPSO shows the same number of clusters as the number of nodes due to the small transmission range, and this decreases gradually downward to 29 as the transmission range is increased. In CLPSO the trend is same. For CACONET the graph shows 49 clusters initially which lowers to 15 when the transmission range is increased.

#### Number of Clusters vs Network Nodes

**Fig 7:**
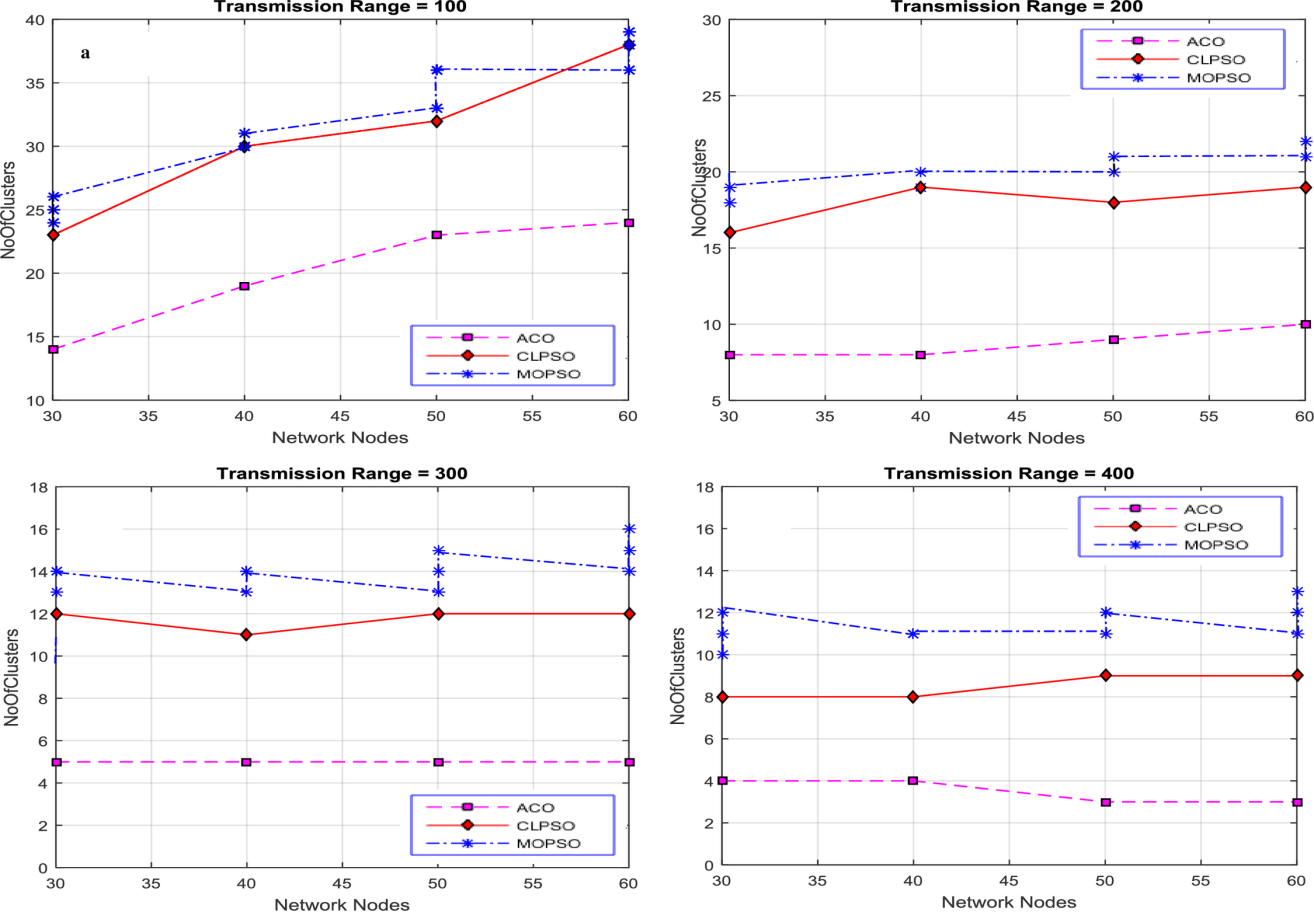
Network nodes vs number of clusters in CACONET, MOPSO and CLPSO in 1 km × 1 km grid size with transmission range varying from 100 m to 400 m.

The number of nodes in a network is varied from 30 to 60 and the transmission range was set to 100, 200, 300 and 400 to conduct the experiments for finding the number of clusters against the number of nodes. Results were produced by varying the grid size from 1 km × 1 km to 4 km × 4 km, as shown in Fig 7.

The results in Fig 7 are produced by fixing the grid size to 1 km × 1 km and using the following transmission ranges 100, 200, 300, and 400. Based on the performance of the three algorithms (MOPSO, CLPSO and CACONET), by keeping the transmission range constant and increasing the number of nodes, it is evident that the transmission range increases as the number of clusters decreases. Fig 7(C) shows that for CACONET the number of clusters remain same for all network nodes. The proposed algorithm works better than the other algorithms in terms of the average number of clusters. This shows the robustness and flexibility of the algorithms in terms of the parameter setting. Fig 7(D) shows that CACONET produces four clusters initially, but with 60 nodes there are three clusters. By analyzing these results it is observed that CACONET performs better in dense traffic areas.

**Fig 8:**
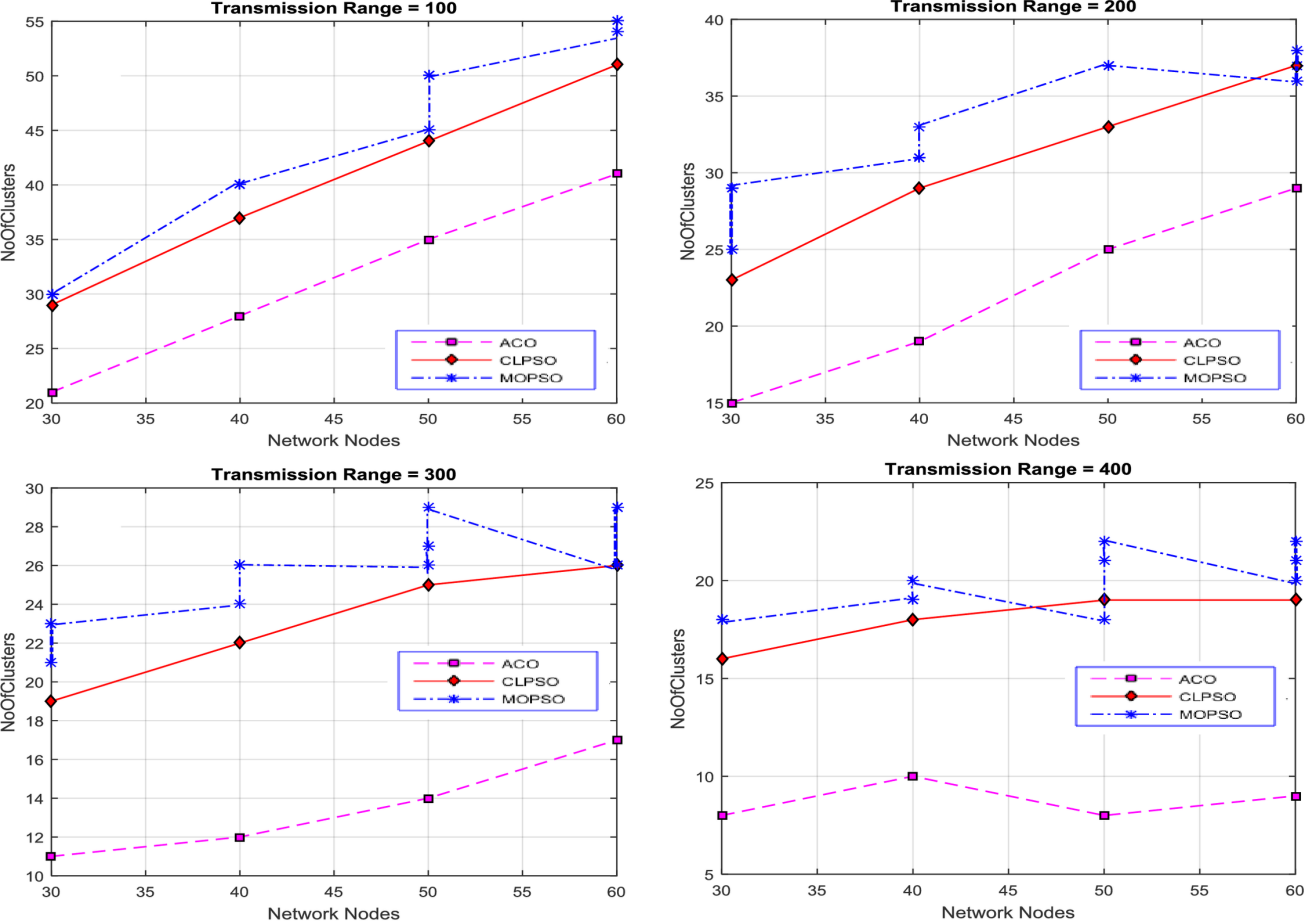
Network nodes vs number of clusters in CACONET, MOPSO and CLPSO in the 2 km × 2 km grid size with transmission range varying from 100m to 400m.

**Fig 9:**
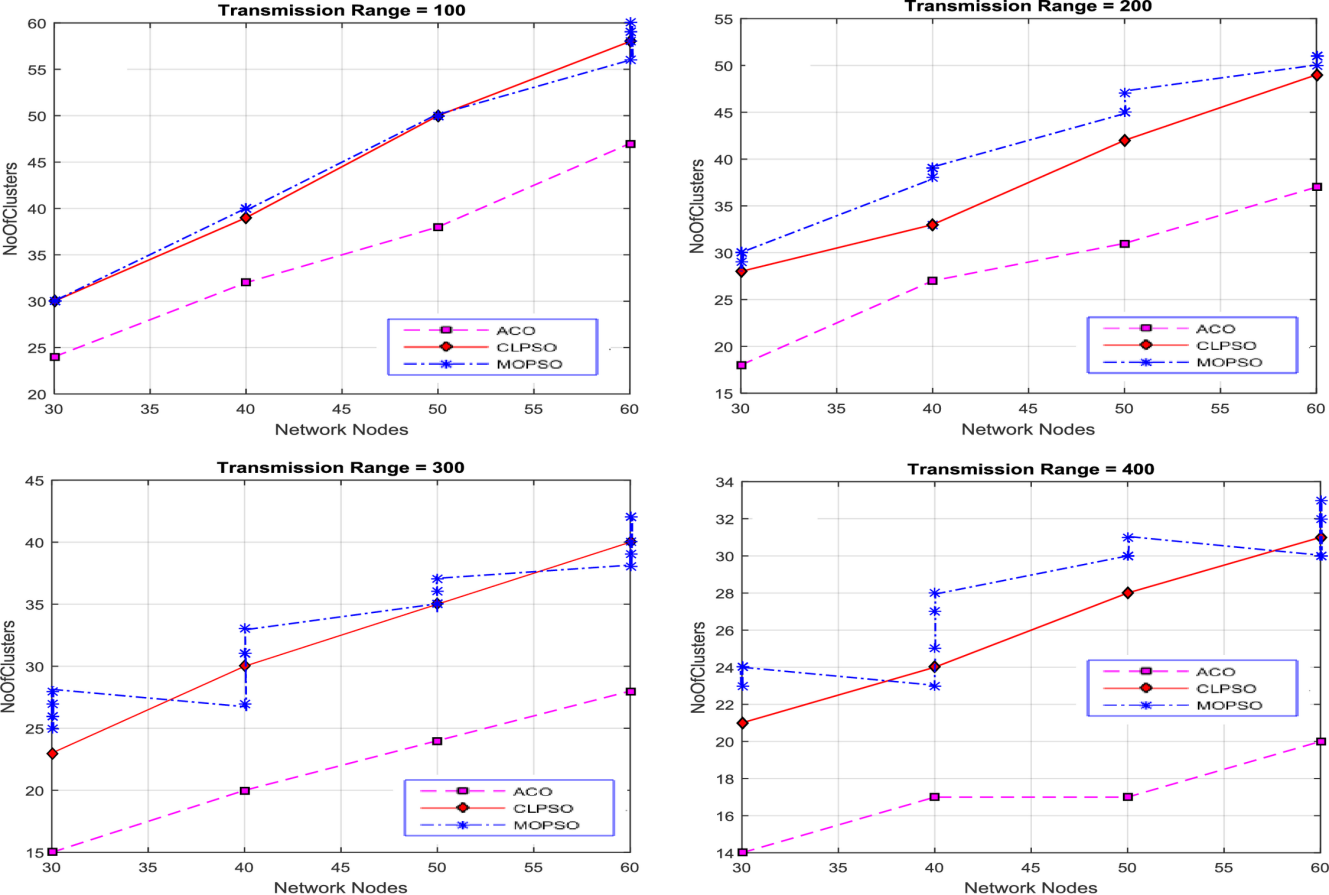
Network nodes vs number of clusters in CACONET, MOPSO and CLPSO in 3 km × 3 km grid size with transmission range varying from 100m to 400m.

Then the grid size is increased to 2 km × 2 km as shown in Fig 8. By evaluating the overall results of MOPSO, CLPSO and CACONET, it is determined that CACONET gives better solutions. Fig 9 shows the results for a grid size of 3 km × 3 km, and the transmission ranges 100, 200, 300, and 400. If we compare Figs 8 and 9, we observe that as the grid size increases, the number of clusters also increases, which shows the direct relation of the network size with the number of clusters.

**Fig 10:**
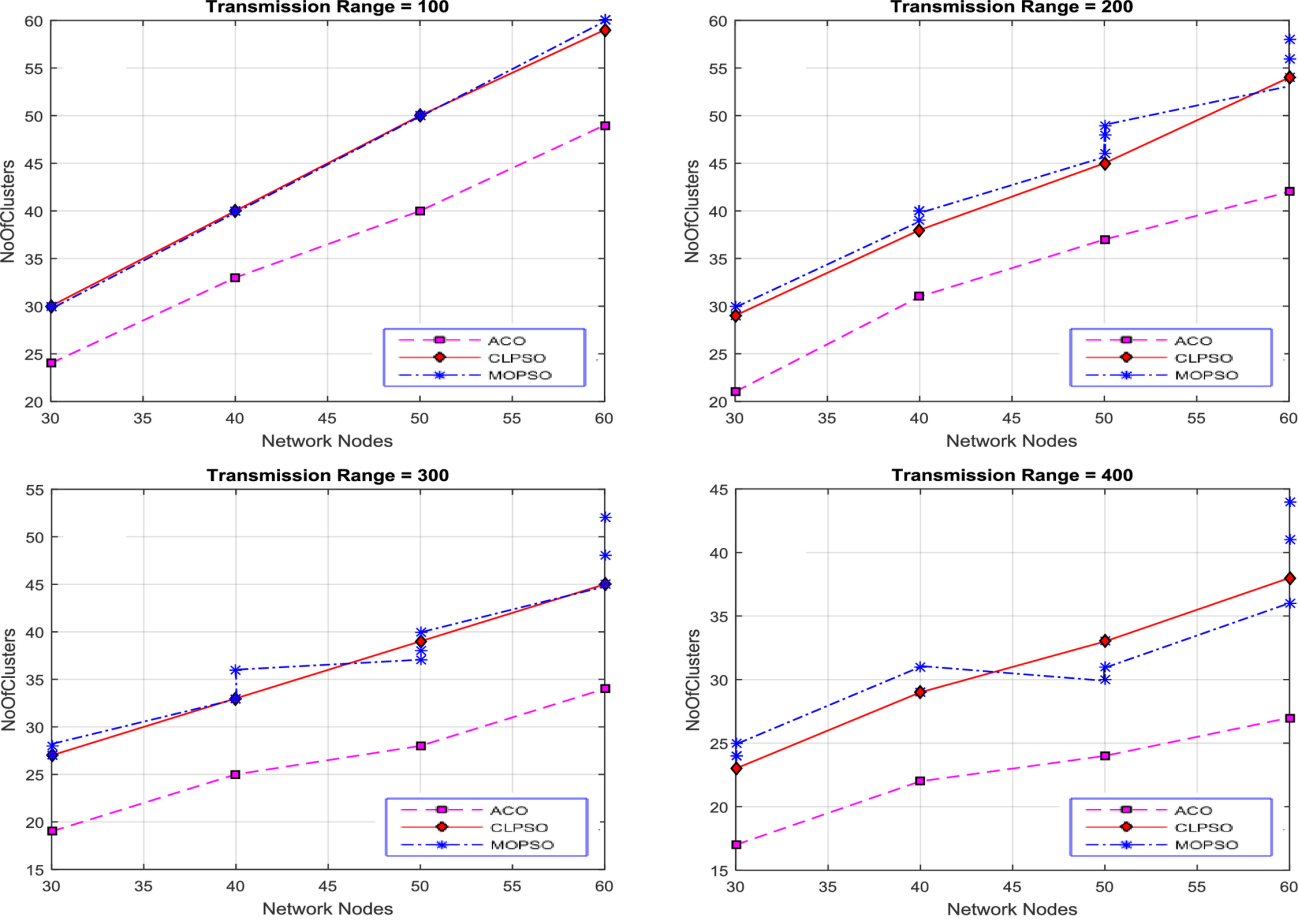
Network nodes vs number of clusters in CACONET, MOPSO and CLPSO in 4 km × 4 km grid size with transmission range varying from 100m to 400m.

Fig 10 shows the results in the case of the 4 km × 4 km grid size with transmission ranges of 100, 200, 300 and 400. The grid size is directly proportional to the distance between nodes. As the grid size increases, the distance between the nodes also increases, which leads to the isolation of the nodes from each other. If all nodes are isolated from each other then all the algorithms must produce the maximum number of clusters. By observing Fig 10(A), 10(B) and 10(C), it is evident that MOPSO and CLPSO produce almost same number of clusters, whereas CACONET generates much better results. In Fig 10(D), when there are 60 nodes in the network, CACONET produces ((38–26) / 38) × 100 = 31% less clusters than the other two algorithms.

#### Number of Clusters vs Grid Size

**Fig 11:**
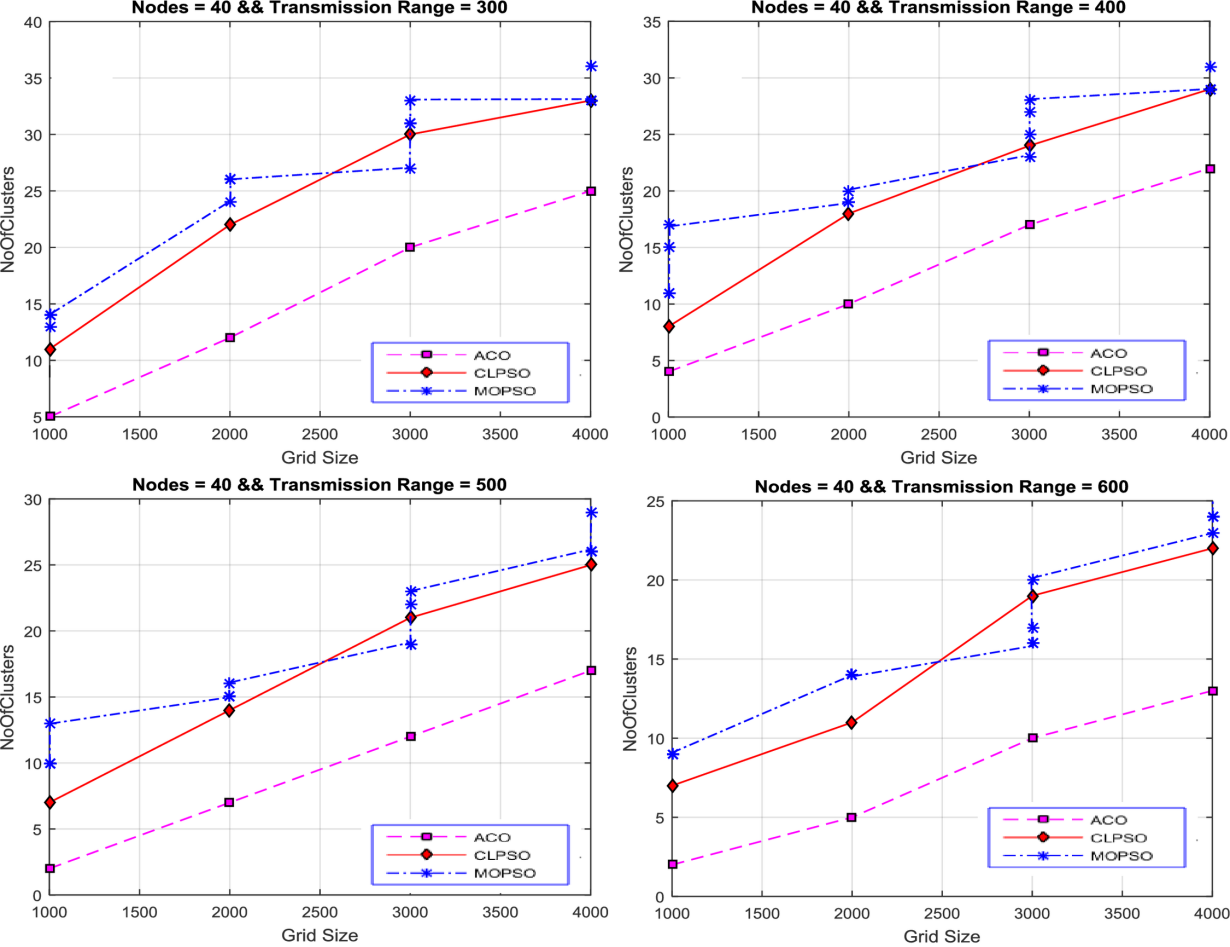
Number of clusters vs grid size in case of CLPSO, MOPSO and CACONET when node = 40 and transmission range varies from 30 to 60.

In Fig 11 the relationship between different grid sizes and the number of clusters is displayed. The number of nodes are fixed at 40 and the transmission range is varied from 300 m to 600 m. Fig 11 shows that the grid size is inversely proportional to the number of clusters because in a large grid size the nodes are more scattered, and therefore a greater number of clusters are required to cover the entire network and vice versa. Manifestly CACONET provides fewer clusters compared to other algorithms, which leads to efficient clustering. Moreover it is determined that CACONET performs better in dense environments.

### Conclusion and Future Work

This paper presents a detailed analysis of multi-objective evolutionary algorithms in VANETs. In the proposed scheme, the node clustering is done efficiently, and near optimal solutions are generated by the proposed algorithm. This makes it best suited among the three algorithms for employment in VANET clustering in the experiments. By minimizing the total number of clusters in the network, the packet routing cost is minimized. Due to the evolutionary capability of these algorithms larger search spaces can be processed, and objective function values can be adjusted dynamically. The flexibility and effectiveness of the approach are exhibited with the help of simulated results. Result comparisons with other well-known algorithms (MOPSO and CLPSO) are also presented here. The optimal number of clusters is found with the help of the proposed CACONET algorithm. Researchers can enhance the list of objectives and make the number of nodes dynamic in future to extend this work. Other evolutionary algorithms can also be implemented, for instance, the Gray Wolf Optimizer, for further extensive comparative studies.

## Supporting Information

S1 File Figure file quality report generated by PACE. (PDF)

## Abbreviations

The following abbreviations are used in this manuscript:

ACO: Ant Colony Optimization
CACONET: Clustering algorithm based on Ant Colony Optimization (ACO) for VANET
CH: Cluster heads
CLPSO: Comprehensive Learning Particle Swarm Optimization
CN: Cluster nodes
DSRC: Dedicated Short Range Communications
ED: Euclidean distance
GWO: Gray Wolf Optimizer
MANET: Mobile ad hoc networks
ITS: Intelligent Transportation Systems
MOP: Multi-objective optimization problem
MOPSO: Multi-Objective Particle Swarm Optimization
PSO: Particle swarm optimization
VANET: Vehicular ad hoc networks
WCA: Weighted clustering algorithm
