# Supplementary material for: CACONET: Ant Colony Optimization (ACO) Based Clustering Algorithm for VANET
Source: PLoS One. 2016 May 5;11(5):e0154080. doi: 10.1371/journal.pone.0154080 (PMC4858224; doi:10.1371/journal.pone.0154080)
Supplement: S1 File — (PDF) [file pone.0154080.s001.pdf]

PLOS

Figure file quality report: 2016-03-31				
Original Filename	PACE Filename	Status	Error Detail(s)	PACE Adjustments
Fig1		✓	• No Error	
Fig2		✓	• No Error	
Fig3		✓	• No Error	
Fig4		✓	• No Error	
Fig5		✓	• No Error	
Fig6		✓	• No Error	
Fig7		✓	• No Error	
Fig8		✓	• No Error	
Fig9		✓	• No Error	
Fig10		✓	• No Error	
Fig11		✓	• No Error	
